# A mixed methods study to understand patient expectations for antibiotics for an upper respiratory tract infection

**DOI:** 10.1186/s13756-016-0134-3

**Published:** 2016-10-20

**Authors:** Christina Gaarslev, Melissa Yee, Georgi Chan, Stephanie Fletcher-Lartey, Rabia Khan

**Affiliations:** 1 University of New South Wales, Centre for Primary Health Care and Equity, Level 3, AGSM Building, Sydney, NSW 2052 Australia; 2 Oslo kommune Helseetaten, Oslo, 0182 Norway; 3 NPS MedicineWise, Evaluation, Sydney, NSW 2010 Australia; 4 Blue Planet research and consulting, 8 highland Crescent, Earlwood, Australia; 5 South Western Sydney Local Health District, Population Health, Liverpool, NSW 2170 Australia

**Keywords:** Antibiotics, Upper respiratory tract infection, Primary care, Inappropriate prescribing

## Abstract

**Background:**

Antimicrobial resistance is a public health challenge supplemented by inappropriate prescribing, especially for an upper respiratory tract infection in primary care. Patient/carer expectations have been identified as one of the main drivers for inappropriate antibiotics prescribing by primary care physicians. The aim of this study was to understand who is more likely to expect an antibiotic for an upper respiratory tract infection from their doctor and the reasons underlying it.

**Methods:**

This study used a sequential mixed methods approach: a nationally representative cross sectional survey (*n* = 1509) and four focus groups. The outcome of interest was expectation and demand for an antibiotic from a doctor when presenting with a cold or flu.

**Results:**

The study found 19.5 % of survey respondents reported that they would expect the doctor to prescribe antibiotics for a cold or flu. People younger than 65 years of age, those who never attended university and those speaking a language other than English at home were more likely to expect or demand antibiotics for a cold or flu. People who knew that ‘antibiotics don’t kill viruses’ and agreed that ‘taking an antibiotic when one is not needed means they won’t work in the future’ were less likely to expect or demand antibiotics. The main reasons for expecting antibiotics were believing that antibiotics are an effective treatment for a cold or flu and that they shortened the duration and potential deterioration of their illness. The secondary reason centered around the value or return on investment for visiting a doctor when feeling unwell.

**Conclusion:**

Our study found that patients do not appear to feel they have a sufficiently strong incentive to consider the impact of their immediate use of antibiotics on antimicrobial resistance. The issue of antibiotic resistance needs to be explained and reframed as a more immediate health issue with dire consequences to ensure the success of future health campaigns.

**Electronic supplementary material:**

The online version of this article (doi:10.1186/s13756-016-0134-3) contains supplementary material, which is available to authorized users.

## Background

Antibiotic resistance is a serious and growing problem, caused in part through overuse and misuse of antibiotics [1]. There is considerable evidence of sub-optimal use of antibiotics in the community. This includes prescribing and use for inappropriate conditions, and use of inadequate treatment courses and sub-therapeutic doses [2–4]. It has been estimated that 20–50 % of all antimicrobial use is inappropriate [5] and Australia contributes to the problem by being one of the largest antibiotic consumers in the world [6]. One of the contributing factors is the inappropriate prescribing of antibiotics for non-specific upper respiratory tract infections (URTIs) [3, 7–9]. Patient/carer expectations have been identified as one of the main drivers for inappropriate antibiotics prescribing by primary care physicians [10–12].

Studies suggest prescribers are influenced by a desire to maintain or establish positive relationships with patients, and prescribe according to their perceptions of patient expectations [7, 13]. Patient misunderstandings can also lead to suboptimal use of antibiotics, such as using short courses and sub-therapeutic doses. Previous studies have found low levels of understanding and common misconceptions about antibiotics among lay people [8–11].

Low education levels, age, low socioeconomic status and ethnicity was found to be associated with variations in knowledge, attitudes and behaviours regarding antibiotic use [5, 13, 14]. Consumers who had a lower income, lower education level, ethnicity, self-reported history of asthma or chronic lung disease and previous antibiotic prescriptions for cough were more likely to believe that antibiotics were always or usually helpful for their condition [14].

Several studies indicated that a high proportion of patients (ranging from 33.5 to 53.1 %) expected an antibiotic prescription for a URTI [7, 15]. Expectations from patients varied depending on symptoms; with earache, fever and sore throat the most common symptoms which led to an antibiotic expectation [7, 15]. Respondents expected antibiotics particularly if they had symptoms for a longer time, were severe or they had other health problems [7]. Parents also expected an antibiotic if their child was previously prescribed an antibiotic for a similar URTI [5]. Perceptions of side effects of antibiotics did not influence expectations for antibiotics [15]. There have been no studies in the Australian context that have looked at antibiotic expectations by patients for an URTI.

In Australia, NPS MedicineWise plays a role in developing health education and campaigns for health professionals and consumers. In 2012, NPS MedicineWise implemented a five-year program to encourage prudent use of antibiotics in the community. Continuous evaluation of this program shows that although there is a high overall knowledge of antibiotic resistance, inappropriate prescribing remains high. There is a need to understand the factors and circumstances that influence patient expectations and demands in the Australian context to develop future public health campaigns. The aim of this study was to understand who expects an antibiotic for an URTI from their doctor and the reasons underlying it.

## Methods

We used a sequential mixed methods approach to understand the characteristics of patients who expect antibiotics for an URTI and the underlying reasons for this expectation. The preliminary phase used a cross sectional survey to estimate the proportion and the characteristics of the population that expect an antibiotic from their doctor for an URTI. The second phase employed focus groups with a purposefully selected sample in an effort to further explore common themes related to expectations for an antibiotics and socio-cultural norms as well as possible communication messages. The use of a mixed methods approach strengthened the validation of the study.

### Quantitative method

The NPS MedicineWise National Consumer Survey is an annual cross-sectional survey conducted with a random sample from the Australian population to assess general public knowledge and awareness of a range of quality use of medicines and medical tests issues. For this study, the results from the survey conducted between April and May 2014 were used. The survey was conducted using an online panel. Invitation emails were sent to a selection on the external panel with a link to the online survey. This process was repeated over five batches until sample size was reached. A random sample of 1,509 Australian consumers aged 16+ years were selected proportionate to geographic location. Quotas were used to ensure representative distribution across geographic locations, gender and age categories.

The survey consisted of 22 questions about health and medicines. Respondents were asked questions about demographics and medicines they take, and then were asked several questions about their knowledge, beliefs and expectations for an antibiotic. The outcome of interest was self-reported expectation for an antibiotic from a doctor if presenting with a cold or flu. The survey questions are available in Additional file 1. The data was weighted to match age and gender proportions of the Australian population. Descriptive analysis and logistic regression analysis was performed using the statistical software program SPSS version 22.

### Qualitative method

Four semi-structured focus group interviews were conducted in August 2014. Focus groups were conducted with groups that were more likely to ask for antibiotics in the quantitative survey results and those identified by general practitioners as more ‘demanding patients’ in a previous study. The discussion guide is available in Additional file 2. The aim was to explore the social and cultural norms surrounding their expectations for antibiotics and understand possible communication strategies to decrease patient demand. We aimed to recruit five to seven people for each focus group; all had been prescribed antibiotics for themselves or their children in the last 6 months by their general practitioner (GP). The groups were mothers with young children under six years of age, low socioeconomic group (household income of less than $70,000), long term migrants (lived in Australia for more than twenty years or born in Australia) who speak either Arabic or Chinese at home (Table 1). The focus groups were conducted in Sydney. Each focus group lasted approximately 2 hours and with participants’ consent, the sessions were audiotaped, videotaped, and transcribed. We compared themes within and across the groups and prioritised issues generating the most discussion, either as views expressed by a majority of participants or minority views that generated much discussion.

## Results

### Quantitative

The response rate for the survey was 22 %. Table 2 displays baseline characteristics of the respondents compared to the general population. The survey participants matched the general population for age and gender. The survey was under represented by culturally and linguistically diverse groups. A smaller proportion among the survey respondents (9 %) rated their health excellent compared with the general population (20 %).

**Table 1:** Characteristics of the qualitative focus group respondents (*n* = 40)

Focus Group	TARGET PROFILE	GENDER	LIFESTAGE/AGE	LOCATION
Group 1	Parents with children between the ages 6 and 12 years	Female	Mix of life stages and age 25 to 60 years	Sydney CBD
Group 2	Parents with at least one child 5 or under (4 were first time parents)	Female		Western Sydney
Group 3	Low SES • Annual household income to be less than $50 k • Highest level of education High School or TAFE	4 x Female 4 x Male		Western Sydney
Group 4	Long term migrants who speak Arabic at home	4 x Female 4 x Male		Sydney CBD
Group 5	Long term migrants who speak Chinese at home	4 x Female 4 x Male		Sydney CBD

**Table 2:** Characteristics of the quantitative survey population (*n* = 1509) compared with Census 2011 for Australia

Characteristic	Responders *n* (%)	Australia %
Males	731 (48.4)	49.4
Age, years		
16–24	234 (15.5)	14.2
25–54	792 (52)	41.8
55–74	363 (24.1)	19.2
75+	120 (8.0)	6.4
Spoke a language other than English at home	176 (11.7)	23.2
Experienced state of health		
Excellent	136 (9.0)	20.4
Very good	547 (36.3)	35.5
Good	562 (37.3)	29.9
Fair	203 (13.4)	10.3
Poor	61 (4.0)	3.9
Education- highest achieved		
University or other tertiary institute degree	477 (31.6)	26 %

**Table 3:** Respondents beliefs about antibiotics (*n* = 1509)

Statement	% Correct	% Incorrect
Antibiotics kill bacteria	70.0	6.5
Bacteria can become resistant to antibiotics	84.1	2.9
Antibiotics kill viruses	43.6	33.0
Taking antibiotics when I don’t need them means they are less likely to work in the future	73.7	8.4

**Table 4:** Findings from the focus group

	Low socioeconomic group	Long term migrants who speak Arabic at home	Mothers with young children group	Long term migrants who speak Chinese at home
Antibiotics IS…	*What WORKS. Cheaper than other Cold and Flu medications.*	*Most POTENT medication (‘magic elixir’). We are OBSESSED with it.*	*A GOD-send especially for the KIDS. I can’t cope without it.*	*For serious Bacterial infection. You can build up resistance, but I prefer to take it when I ‘have to’.*
I’ve heard/	• My GP keeps saying take it only when you get worse	• Prevention better than cure!	• Young children recover faster with antibiotics	• In China, Singapore, Hong Kong and Taiwan doctors will administer antibiotic intravenously at first sign of any illness) • People who use antibiotics frequently when they are not seriously ill develop antibiotic resistance • Of super bugs, resistant to antibiotics
I believe…	• It only works on bacteria or virus… germs in general (like Ebola) • You shouldn’t take it all the time (but I don’t really understand why? - I’ve been taking it regularly for 20 years and it’s worked!)	• Of antibiotic resistance, the doctors will put you onto stronger doses or change the brand/type of antibiotics • Some people I know is now resistant, Amoxyl doesn’t work anymore on them	• Kids can develop pneumonia from the common cold if left untreated with antibiotics • Of antibiotic resistance, but it’s alright because I only use it when we are very sick	
I EXPECT Antibiotics when…	• Winter months when lots of people get sick with cold and flu • I can feel ‘it’ coming (scratchy sore throat, if I don’t nib it in the bud, it’ll take me 2 weeks to recover)	• I KNOW my child/my body is going to get worse (I know my body or child’s body better than the GP)	• Kids are sick for more than 3 days • Kids get the same illness (like croup) all the time • Especially winter months, daycare is filled with sick kids and germs • Bacterial infection	• I have a serious illness or bacterial infection • GP says I have a 60–60 % chance my illness could be caused by bacteria or virus • I’ve waited for more than an hour at the GP
Therefore I ASK for Antibiotics BECAUSE…	• I KNOW it works (as it has many time in the PAST!) • They put me on the ‘stronger dose’/changed the brand (they can keep introducing new ‘brands’ anyway)	• I know if I nip it in the bud with antibiotics straight-away, my child will recover faster than without it • I feel more SECURE when I take it	• My child can’t recover (quickly) without it • I can’t cope with all 4 of my kids sick at home (domino effect) • I need to get back to work (and can’t take so many days off to look after sick children)	• I want a TANGIBLE BENEFIT to compensate for my time (in the waiting room) • Security just in-case I get worse, I don’t want the inconvenience of re-visiting the GP
When the GP gives me a script and say fill it later, I…	• Filled it straight away! Why would I wait until it gets worse?			• I wait and monitor my condition and will only fill the antibiotic script if I don’t get better
Others like me…	• Everyone takes it more in winter • They take a few and stop when they get better • Buy them in bulk from overseas	• My family and friends take antibiotics even more than I do • My family takes it like multi-vitamins • Stop when I feel better/ Save left-over doses for emergency	• Mums who don’t work can afford to stay at home with their sick kids, so they probably don’t need to give their kids antibiotics as often as I do	• Buys in bulk from overseas and self-medicate for conditions such as back pain
I WORRY about…	• Thrush, diarrhoea • I’m now resistant to a few of the antibiotics • Expense	• Thrush, diarrhoea	• Kids have tummy upsets (constipation or diarrhoea) • Killing off all the good bacteria • Antibiotic resistant super bugs	• Killing off all the good bacteria • Antibiotic resistant super bugs • Being prescribed antibiotics unnecessarily

#### Knowledge and beliefs

**Fig. 1:**
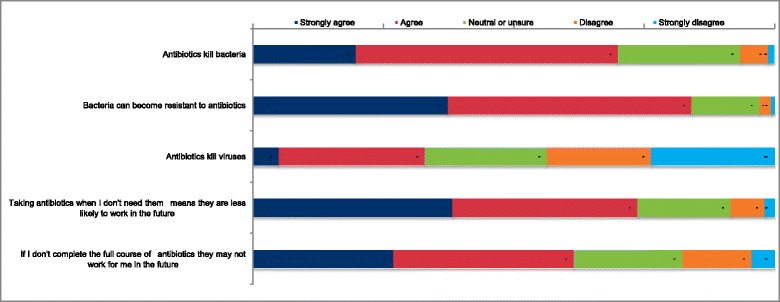
Respondents reported knowledge and beliefs of statements about antibiotics (*n* = 1509)

Most respondents, 70 % (*n* = 1057), agreed that antibiotics kill bacteria. However, 33 % (*n* = 497) also agreed that antibiotics kill viruses (Fig. 1). Of all respondents only 36.5 % (*n* = 552) correctly identified antibiotics as effective against bacteria and not viral infections.

Overall 69.8 % of respondents (*n* = 1053) reported having heard of the term antibiotic resistance, whilst 10.2 % were unsure (*n* = 154). Age and educational level were associated with having heard of antibiotic resistance (*p* < 0.001). A higher proportion (*n* = 1269, 84.1 %) correctly agreed or strongly agreed with the statement ‘Bacteria can become resistant to antibiotics’ (Fig. 1, Table 3), and this was associated with age and educational level (*p* < 0.05).

Among respondents 73.7 % (*n* = 1112) were aware that taking antibiotics when they don’t need them means that they are less likely to work in the future (Fig. 1). This awareness increased with age. Similarly, 61.5 % (*n* = 928) agreed that they have to take a full course of antibiotics or they may not work in the future, and the proportion agreeing increased with age.

#### Expecting antibiotics

Only 19.5 % (*n* = 284) of respondents reported that they expect their doctor to prescribe antibiotics if they have a cold or flu, with the most significant predictors including speaking a language other than English at home (*p* < 0.002) and younger age groups (*p* < 0.028). Of these patients, not all voiced their expectation for a prescription to their doctor. Overall only 16.9 % (*n* = 254) of respondents say they would directly ask their doctor for antibiotics when they have a flu or cold. However the proportion is higher among those that speak a language other than English at home (*p* < 0.01). The proportion of respondents who would directly ask their doctor for antibiotics is positively associated with educational level (*p* < 0.048) and age. In terms of knowledge, people who knew antibiotics didn’t kill viruses and agreed that taking an antibiotic when one is not needed means they won’t work in the future were also less likely to expect or demand antibiotics (*p* = 0,000).

### Qualitative

In total, 21 people participated in the four focus groups. Each group consisted of 5 or 6 participants; all were prescribed antibiotics for themselves or their children in the last 6 months by their GP. The results are presented under four main themes: knowledge and understanding of antibiotics and antibiotics resistance, time investment, miscommunication with the GP, and beliefs about consequences of taking antibiotics inappropriately. Table 4 summarises the main themes by group. 

#### Knowledge and understanding of antibiotics and antibiotics resistance

Most focus group participants showed considerable gaps in their knowledge about antibiotics and how resistance developed. Furthermore, respondents in the focus groups all stated that they demanded antibiotics from their GP even after the GP initially did not prescribe antibiotics. Their reasons for demanding antibiotics for a cold or flu varied between the groups. However, there was a consensus that the individual was in the best position to decide if an antibiotic is needed for themselves or their children, hence the expectation and sometimes demand for antibiotics. Most of the participants in all of the groups perceived of antibiotic resistance as a change in the human body rather than in bacteria.

The low socioeconomic group showed a low level of understanding of how antibiotics work for what conditions, and the difference between viral and bacterial induced illnesses. No one in the group knew what antibiotic resistance was but they all had heard of the term before the focus group. This group believed that antibiotics in general are more efficacious and cheaper compared to over the counter cold and flu medication and they did not understand why they would not ask for antibiotics if they were ill. Furthermore, they expressed that if the doctor withheld antibiotics, it was not because of medical reasons but rather that the doctors disliked antibiotics or wanted to save the government money.

*“I had to ask for it. I said nothing’s working and I’ve done everything, and so I said I want it. Please can I get something stronger that you can prescribe? Just talking amongst my friends they all say their doctor does that too. Maybe it’s a code that they’ve been told not to prescribe any medication. If you go there determined and you say I need something prescribed to me now, they’ll give it to you.” (Respondent, low socioeconomic group)*

The Arabic speaking group also showed a low understanding of antibiotics and thought antibiotics were for general illnesses. There was also a low level of understanding of antibiotic resistance in that the group, who also strongly believed that the body becomes resistant and there will always be other stronger antibiotics for them to switch to later if they developed resistance.

*“Antibiotics, Love it. Shame, you have to go to a doctor to get prescribed them, you can’t just go to the pharmacist. Because it kills things very quickly. When you see them go green then you know they need antibiotics. Because you know they have an infection. I run to the doctor because I know they need antibiotics and that is what will treat them. In 2 days they will be fine.”* (*Respondent, Arabic speaking group)*

*“My mother, she’ll wake up, and if she feels sick she takes antibiotics. She is always at the pharmacy, and has a whole cupboard full. She will take it when there is nothing seriously wrong with her, as in no temperature. And she gives me a hard time when I don’t give my kids antibiotics.”* (*Respondent, Arabic speaking group)*

All the participants in the mothers with young children group knew that antibiotics killed bacteria only and had some understanding of antibiotic resistance. However, they expected, and at times demanded, antibiotics for their children, especially if their child had a similar illness previously or the child was sick for longer than expected. There was also a common belief in this group that an antibiotic prescription would prevent the other children falling ill and will shorten the duration of the child’s illness.

*“They (antibiotics) kill the germs and make you feel better. They give you your strength back a lot quicker. You’re not sick for too long. It cuts the length of the germ that’s in your body so you are re-gaining your health a lot quicker. You are feeling a lot better about yourself and you’re not taking too much time off work. If the kids aren’t sick they aren’t missing out on school and they aren’t having to catch up. There are a lot of benefits to it …” (Respondent, mothers with young children group)*

*“If you take it too often then you become resistant to it and there’s always that thought in my mind that I might really need it one day. You kind of do need to power on and so that’s in the back of my mind.”* (*Respondent, mothers with young children group)*

All the participants in the Chinese speaking group knew that antibiotics killed bacteria only and had a reasonable understanding of antibiotic resistance. They expected antibiotics if they had a serious illness or there was a chance of a bacterial infection. For this group, the belief that their illness is serious and they have a chance of developing an additional infection were their main reasons for expecting an antibiotic.

*“The last time was a year ago, I had a really, really bad throat infection. I went to see a doctor to check it’s a throat infection and she said yes. This is a doctor that tends not to prescribe antibiotics. You talk about it all with your friends and the general belief is that antibiotics would kill the bad bacteria but it might also take away your good bacteria as well but at the same time you need to fix the problem. A colleague of mine would say while taking antibiotics it’s good to take other stuff like yoghurt and for her it works but for other people it may not work. So I had a very bad throat infection and I had a temperature and so I went to see the same doctor and she said my throat was bad. It could be bacterial but it could be viral, she said if it is causing you discomfort you may’s well take it because there is a 50 % chance that you could get fixed which she prescribed to me and I took it and it was only a weak one and it did work. It got rid of my throat infection and it did work and of course it could work on others but you eat nutritious food, pick up information about yoghurt and stuff like that. For me it did work, there are times when I’m sure I need antibiotics but I’d rather be cautious and go without it.”* (Respondent, Chinese speaking group)

#### Time investment

A script for antibiotics was considered a necessary tangible benefit to compensate for the time and effort invested to visit the doctor and waiting in the waiting area. This was one of the reasons patients demanded antibiotics as it meant the patient could avoid the inconvenience of visiting the doctor again if their condition deteriorated. This theme was common across the four groups.

*“If you go to a doctor you want to walk away with something. It’s not just advice and to go home and rest. I don’t have to see the doctor to do that.” (Respondent, Chinese speaking group)*

“*Some doctors push antibiotics all the time, some refuse to give it. I don’t really understand the reasons. They always say it’s viral. It frustrates me I have to tell my GP I’ve waited for an hour to see him and I don’t want to have to come back again in a few days and wait again! I really need a script for myself or my kids. They will give me a script anyway, and sometimes say don’t fill it unless you get worse. To be honest, I fill it straight away. Why take the risk of getting worse? I want myself or my kids to get well quickly!” (*Respondent, *Arabic speaking group)*

#### Miscommunication with the doctor

We found in all four groups communication gaps between GPs’ advice and participants’ understanding. This was evident mainly in two areas: the viral nature of their condition and delayed prescribing.

Consumers were confused about what symptoms required antibiotics and what a viral condition was. Most participants reported that they couldn’t understand why their doctor wouldn’t prescribe them antibiotics and suggested that their condition was viral. There was also skepticism among the participants that because the doctor wasn’t able to diagnose them appropriately; hence the diagnosis with a viral condition.

*“The GP doesn’t know what illness I have, I have to insist on antibiotics.” (Respondent, low socioeconomic group)*

‘W*hen the GP doesn’t know what is wrong with me or my child they just say ‘it’s viral’.” (Respondent, mothers with young children group)*

There was further confusion among the groups regarding delayed prescribing around the reason for the delay or waiting period. All reported that they filled the prescription straight away and couldn’t understand the logic behind the request to delay.

Examples of responses given when GPs tell them not to fill the prescription until they’re worse or in a few days:

*“No I fill the script straight away because I have always had trouble with my throat.” (Respondent, mothers with young children group)*

*“To be honest, I go straight to the chemist. I don’t listen to him because I want to get out of the terrible sickness. I get the antibiotics cause I know it helps me.” (Respondent, Arabic speaking group)*

#### Beliefs about consequences of taking antibiotics inappropriately

All the participants mentioned side effects as a consequence of taking antibiotics but this didn’t deter them. No one mentioned antibiotic resistance until prompted and didn’t realise that they personally could contribute or be affected by the issue. They perceived that personally they and their families had a low or negligible risk from antibiotic resistance. The issue of antibiotic resistance was an issue for future generations or an issue in developing countries and they themselves or their families would never be affected by its consequences.

*“I was thinking of the person over-using and not the community. It’s more like drugs. That person over used (it) and so he is going to get the problem (antibiotic resistance), it’s not everyone else is going to get the problem.” (Respondent, Chinese speaking group)*

*“In future, if (antibiotics) doesn’t help anymore. You need to take a stronger pill. But that’s in the future; you have to focus on this point in time.” (Respondent, Chinese speaking group)*

*“I know about it (antibiotic resistance) but antibiotics work for me. Is there an alternative for antibiotics? But as long as there is no other option and it works for me, I’m going to keep taking it.” (Respondent, low socioeconomic group)*

*“If it’s (antibiotic resistance) true I wonder if it’s worldwide or Australia wide. I think it would be worldwide. But in third world country they don’t have antibiotics so they would have basic bugs but they would be stronger too.” (Respondent, mothers with young children group)*

## Discussion

This sequential mixed methods approach combines survey and focus group data to understand the characteristics of patients who expect antibiotics for an URTI and the reasons underlying their expectation. Despite good levels of knowledge with regards to the effect of antibiotics and the risks of developing antibiotic resistance, we found 19.5 % of surveyed consumers reported that they would still expect the doctor to prescribe antibiotics for a cold or flu and 16.9 % would ask a doctor to prescribe antibiotics. The reason for this remains unclear and requires further study, but anecdotal evidence suggests that this may be associated with a long standing culture of not leaving the doctor empty handed.

The survey results indicated that people under 65 years of age, never attended university/technical college and who speak a language other than English at home were more likely to expect or demand antibiotics for a cold; although this was lower than in other studies. This could be due to the significant underrepresentation of CALD groups in our survey sample and the slight over representation of respondents who had attended university or technical college. However, this would be an issue affecting other surveys as well.

Three of the four groups had a clear expectation of receiving antibiotics for a cold or flu if presenting to a doctor. The main reasons given for this expectation is antibiotics help them feel better, prevention of potential deterioration of illness, previous successful experience and investment of time and money to consult a doctor. This is in keeping with other studies that found patients/carers lack an accurate understanding of the nature of antibiotics and the development of antibiotic resistance [12, 13, 15–17]. Evidence suggests that many patients are confused about how resistance is developed [15, 16] and may explain why participants in this study did not understand the personal consequences of inappropriate antibiotic use. Patient expectations may therefore be playing a significant role in inappropriate antibiotic prescribing in primary care settings. The evidence suggests that patient expectations are important to general practitioners, and GPs are more likely to prescribe antibiotics when their patients are perceived to be expecting them [18, 19].

There was evidence of misconceptions among patients about what doctors mean. Most respondents felt that if the GP said that their illness was viral it indicated that the doctor didn’t know what was wrong with them and or didn’t want to give them a prescription. One study found that some expectations are shaped by their experience of widespread availability of antibiotics in their home country and that they hold substantial misconceptions about antibiotics use [20].

Misconceptions concerning delayed prescribing and the need to wait before using the prescription were reported in this study and have been described elsewhere [21] Advocates for delayed prescription believe that, in addition to reducing antibiotics consumption, it increases patient empowerment and satisfaction, reduces re-consultations and provide health professionals with a safety net [22, 23]. However, our study is consistent with other studies that have shown that a significant proportion of patients fill the delayed prescription on the day of receiving it [13, 22].

Respondents’ misconception that the human body can become resistant to antibiotics has been reported in other studies [13, 16]. This misconception may explain why focus group participants appeared to be acting in rational self-interest (or in the interest of their children) in their belief that they cannot individually change the problem of resistance, hence any chance of a modest benefit from antibiotic use outweighs the negligible contribution that they could make to the common good by refraining from their use.

The study has several limitations. The consumer survey was based on an online panel and therefore is limited to participants on the panel population. However, sampling methods ensured the participants were representative of the general population. Another potential limitation of the study is that the number of respondents rating their experienced state of health as excellent is lower than the general population. This can indicate that the respondents are more frequently exposed to the health care system and therefore more frequently in need of antibiotics. The response rate was only 22 %, but this is considered typical for online population surveys in Australia [16]. The selection criteria for the focus groups were informed by GPs perception of high demand patients and those in the survey who were more likely to expect an antibiotic. However, the generalisability of the study could have been improved with the inclusion of more consumer groups in the focus groups, including recently arrived migrant groups and further stratification of the current groups by socioeconomic status.

## Conclusion

To our knowledge, this is currently the largest Australian study exploring consumers’ knowledge and expectations for antibiotics when presenting with an URTI. This study has identified several key motivations for why patients expect antibiotics and their lack of understanding of personal consequences of antibiotic resistance that have not been addressed by recent public health campaigns. Several misconceptions amongst patients and their understanding of why GPs do or do not prescribe antibiotics indicate an urgent need to bridge the communication gap.

Primary care physicians will need to be trained to develop better communication strategies for dealing with patients. There is an urgent need for the core message of future public health campaigns to be focused on the personal consequences of taking antibiotics inappropriately and the implications of antibiotic resistance for the general public. Key messages should focus on the immediate and dire repercussions of antibiotic resistance for individuals and their families in the short term.

## Additional files

Additional file 1: Extract: national consumer survey 2014 (DOCX 26 kb)
Additional file 2: Focus Group Discussion Guide (DOCX 18 kb)

## Abbreviations

CALD: Culturally and linguistically diverse
GP: General practitioner
URTI: Upper respiratory tract infections
