# Supplementary material for: A mixed methods study to understand patient expectations for antibiotics for an upper respiratory tract infection
Source: Antimicrob Resist Infect Control. 2016 Oct 20;5:39. doi: 10.1186/s13756-016-0134-3 (PMC5072313; doi:10.1186/s13756-016-0134-3)
Supplement: Additional file 1: — Extract: national consumer survey 2014 (DOCX 26 kb) [file 13756_2016_134_MOESM1_ESM.docx]

**Supplementary Material 1**

**Appendix 1 –** Extract: national consumer survey 2014

**Section 5. Antibiotics**

**Q11**. Please indicate your level of agreement or disagreement with the following statements about antibiotics:

*Please select one answer in each row*

|  |  | Strongly Agree  1 | Agree  2 | Neutral  3 | Disagree  4 | Strongly Disagree  5 | Unsure  6 |
| --- | --- | --- | --- | --- | --- | --- | --- |
| a | Antibiotics kill bacteria | ⭘ | ⭘ | ⭘ | ⭘ | ⭘ | ⭘ |
| b | Bacteria can become resistant to antibiotics | ⭘ | ⭘ | ⭘ | ⭘ | ⭘ | ⭘ |
| c | Antibiotics kill viruses | ⭘ | ⭘ | ⭘ | ⭘ | ⭘ | ⭘ |
| d | Taking antibiotics when I don’t need them means they are less likely to work in the future | ⭘ | ⭘ | ⭘ | ⭘ | ⭘ | ⭘ |
| e | If I don’t complete the full course of antibiotics they may not work for me in the future | ⭘ | ⭘ | ⭘ | ⭘ | ⭘ | ⭘ |

**Q12**. If you had a cold or flu, would you?

|  |  | Yes  1 | No  2 | Unsure  3 |
| --- | --- | --- | --- | --- |
| a | Ask a doctor to prescribe antibiotics for you | ⭘ | ⭘ | ⭘ |
| b | Expect a doctor to prescribe antibiotics for you | ⭘ | ⭘ | ⭘ |

**Q13**. Have you heard of the term **antibiotic resistance**?

| 1 | ⭘ | Yes |
| --- | --- | --- |
| 2 | ⭘ | No [Skip to Q14] |
| 3 | ⭘ | Unsure [Skip to Q14] |

[Ask Q13.1 if YES (code 1) in Q13]

**Q13.1**. How likely are you to discuss **antibiotic resistance** with your family or friends?

|  | | | | | | | | | | |
| --- | --- | --- | --- | --- | --- | --- | --- | --- | --- | --- |
| 0  **Extremely unlikely** | 1 | 2 | 3 | 4 | 5 | 6 | 7 | 8 | 9  **Extremely likely** | 10 |
