# Supplementary material for: A mixed methods study to understand patient expectations for antibiotics for an upper respiratory tract infection
Source: Antimicrob Resist Infect Control. 2016 Oct 20;5:39. doi: 10.1186/s13756-016-0134-3 (PMC5072313; doi:10.1186/s13756-016-0134-3)
Supplement: Additional file 2: — Focus Group Discussion Guide (DOCX 18 kb) [file 13756_2016_134_MOESM2_ESM.docx]

**Supplementary Material 2**

**Appendix 2 – Focus Group Discussion Guide**

**Suggested Focus Group Discussion Guide Flow (1.5 hours):**

1. Introduction, household, livelihood, background
2. Attitude and behaviour towards Antibiotics: WHAT, WHEN, WHERE, WHY, WHY NOT
3. Typical encounter with GPs from patient’s view point
4. Communications regarding Antibiotics
5. Drivers to reducing future requests or acceptance of non-prescription of Antibiotics
6. Closing remarks

**Section1: Introduction, household, livelihood, background (5 minutes)**

Tell me about you…

- Who you live with?
- Hobbies?
- Work?
- Favourite foods? Cultural/language spoken at home? Background?

**Section2: Attitude and behaviour towards Antibiotics: WHAT, WHEN, WHERE, WHY, WHY NOT (20-15 minutes)**

Where do you get information about health and medicines from? LIST ON WHITE-BOARD

- Social media to obtain health information? PROBE: Facebook, Twitter

Tell me about antibiotics…

- What comes to mind? Associations, images, feelings, colours, pictures?
- Any associations with sniffles/cold/flu? UNDERSTAND WHAT AND WHY
  - Viral vs bacteria?

Tell me about the last 2 times you were prescribed antibiotics either for yourself or for your kids…

- What happened?
- What did you say/GP say? LISTEN TO STORIES FULLY. ALLOW RESPONDENTS TO NORM AND BOND.

When do you expect to be prescribed antibiotics?

PROBE SYMPTOMS, DRIVERS TO GET WELL, EMOTIONAL REASSURANCE

MOTHER’S GROUP ALSO ASK:

- Do your children attend daycare centres?
- Have your expectation of antibiotics prescription changed since they started daycare?
  - If so, how?
- Ask for antibiotics more on Friday? How come?

Were there times when you asked for antibiotics and the GP resisted at first?

- What happened?
- What did you say/GP say? LISTEN TO STORIES FULLY. ALLOW RESPONDENTS TO NORM AND BOND.

**Section3: Typical encounter with GPs from patient’s view point (10 minutes)**

Tell me more about when you are at the GP’s office when you were prescribed antibiotics…

- How did you feel? How come?
- Did the GP offer antibiotics?
- Did you ask for antibiotics? Why/why not?

Were there times when you were at the GP’s office when you asked for antibiotics but was **not** at first prescribed by your GP…

- What happened?
- What did the GP say or do?
- How did you feel? How come?
- What did you do? Did you visit another GP to get the antibiotics? Why/why not?

Has your GP given you a prescription of antibiotics and said don’t fill it now, only if you or your child gets worse then fill it?

- What happened? Did you feel it? Why/why not?

**Section4: Communications regarding Antibiotics (10 minutes)**

Tell me more about antibiotics…

- What are all the good things about antibiotics? How come?
- Bad things? How come?
- Where did you hear about this? PROBE ALL SOURCES OF INFORMATION, INFLUENCERS AND INFLUENCES
  - What was the key message?
  - How did it make you feel?
  - Was it relevant to you? Why/why not?
  - Did it persuade you one way or another? Why/why not?

**Section5: Drivers to reducing future requests or acceptance of non-prescription of Antibiotics (15-20 minutes)**

What are all the things that need to happen for people to not request for antibiotics from their GP? LIST ON WHITE-BOARD

FOR MESSAGES, ASK:

- What information needs to be shared?
- To whom?
- In what way?
- How and where would people hear about them?
- What might their reactions be?
  - What would cause people to be more open to these messages?
  - What would cause people to ignore or dismiss these messages?

CHUNK UP DRIVERS INTO THEMES/PLATFORMS IF RELEVANT.

ASK FOR EACH:

- What is this really about, in your own words?
- What is at the very heart of this?
- Would it be persuasive to people?
  - More persuasive to which groups of people? How come?
  - Less persuasive to which groups of people? How come?

**Section8: Closing remarks (5 mins)**

Thank you. We greatly value your input.

THANK & CLOSE.
